# Supplementary material for: Detection of Chronic Blast-Related Mild Traumatic Brain Injury with Diffusion Tensor Imaging and Support Vector Machines
Source: Diagnostics (Basel). 2022 Apr 14;12(4):987. doi: 10.3390/diagnostics12040987 (PMC9030428; doi:10.3390/diagnostics12040987)
Supplement: Supplementary file 1 [file diagnostics-12-00987-s001.zip › diagnostics-1618423-supplementary.pdf]

Supplementary Materials

Methods

1.0 DTI Metrics

Anisotropy is the property represented by the eccentricity of conic sections in three dimensions, each normalized to the unit range, 0.0–1.0. Implicit isotropy is 0.0 and is equally constrained (or unconstrained) in all directions (axes). Anisotropy indicates less than maximal isotropy, which is 1.0. In a diffusion ellipsoid, let λ_1, λ_2, λ_3 represent the three principal eigenvalues of the diffusion tensor, then

$$\text{Mean Diffusivity, } MD = \frac{\lambda_1+\lambda_2+\lambda_3}{3} \ (\text{mm}^2/\text{s})$$

$$\text{Axial Diffusivity, } AD = \lambda_1 \ (\text{mm}^2/\text{s})$$

$$\text{Radial Diffusivity, } RD = \frac{\lambda_2+\lambda_3}{2} \ (\text{mm}^2/\text{s})$$

$$\text{Fractional Anisotropy, } FA = \sqrt{\frac{3}{2}}\sqrt{\frac{(\lambda_1-MD)^2+(\lambda_2-MD)^2+(\lambda_3-MD)^2}{{\lambda_1}^2+{\lambda_2}^2+{\lambda_3}^2}}$$

$$the\ ratio\ of\ AD\ over\ RD, \frac{AD}{RD} = \frac{2\lambda_1}{\lambda_2+\lambda_3}$$

2.0 Motion Correction

The imaging protocol was designed to avoid issues with insufficient data when removing volumes from the concatenated dataset due to the presence of artifacts or motion. Our DTI protocol consisted of acquiring 5 non-diffusion weighted images (b = 0) and 51 diffusion directions (b = 1000 s/mm^2, 56 brain volumes). We inspected our analysis path and found that on average we removed 1–2 volumes from the concatenated DW data for each subject (1–2 volumes out of 112 brain volumes). We had two subjects in which we had to remove 5 volumes. For these

subjects, these volumes were either in the first or the second acquisition. Consequently, we had data acquired at 51 diffusion encoding directions for each subject and avoided corruption of the same diffusion encoding direction within a scan.

3.0 SVM Analyses

We implemented an SVM-based model to distinguish bmTBI from HCs based on linear combinations of multiple diffusion metrics from clusters in multiple white-matter regions, which were identified as described in Section 2.4 of the main text. Supplementary Figures S1b and S2 show a flowchart and visualization of the procedure to construct the classification model. The complete procedure includes one algorithm and three routines: (1) *DTI-based bmTBI classification algorithm*, (2) *routine stepwise SVM feature selection*, (3) *routine SVM cross-validation*, and (4) *routine SVM*. The core concept of the procedure is to try all possible combinations of the DTI metric set and select the optimal combination(s). For each subset of metrics, we select the features for classification by removing the least relevant feature recursively. The descriptions of the above procedures are provided below.

The *DTI-based bmTBI classification algorithm* inputs the set of metrics and features and returns the best subset of metrics and features to classify bmTBI. When running the *algorithm*, we first normalize the features to zero mean and unit-variance, and then we enumerate all possible combinations of the metric set and call *Routine Stepwise SVM Feature Selection* to find the best solution with respect to the given combination of metrics. The solution with the lowest error among the subset of metrics is selected and returned.

Routine stepwise SVM feature selection inputs the feature matrix and outputs the best subset of features with feature weights, the bias and the classification error. For each iteration the process calls *Routine SVM Cross-Validation* to determine the feature weights and bias of SVM

classification and the error with cross-validation with a given subset of features. The routine then deletes the feature with the smallest weight magnitude (i.e., the feature of the least relevance to the classification). The iteration stops when all features are exhausted. The solution of the optimal feature set is reported.

Routine SVM cross-validation inputs the feature matrix and outputs the results of the cross-validation tests. For each iteration, the process randomly selects five samples out and calls *Routine SVM* for a cross-validation test. In this study, we chose to run leave-five-out cross-validation tests ten thousand times and calculated the averaged feature weights, correctness, sensitivity, and specificity, defined as:

$$correctness = \frac{N_c}{N},$$
$$sensitivity = \frac{N_{c.bmTBI}}{N_{bmTBI}},$$
$$specificity = \frac{N_{c.HC}}{N_{HC}},$$

where N, N_c are the number of the testing data and correctly classified data, respectively; the subscripts denoted with bmTBI, and HC represent the data in bmTBI and HC group.

Often, obtaining large datasets for machine learning (ML) in medicine is difficult, however, cross-validation can be used to evaluate the performance of a built model with a small dataset [66]. Cross-validation is a robust technique that provides enhanced confidence of ML classification results through the validation of testing datasets with the model built on training datasets [5]. Thus, combining SVM together with cross-validation can potentially generate a robust model to identify features that best separate bmTBI individuals from HC. In this study, we leave five subjects out (k = 5) in each validation because normally, the segmentation of validation data in small datasets is around 10–15% [67].

Routine SVM is the standard procedure of a linear kernel SVM construction. The fundamental concept of SVM is to seek a separating hyperplane between bmTBI and HC, and the expressions are:

$$w^T x + b \geq 1 \Rightarrow bmTBI,$$

$$w^T x + b \leq -1 \Rightarrow HC,$$

where w is a column vector with each element representing the weight of each feature; x is a column vector with each element representing one feature for one subject; b is a bias term. For the stepwise SVM employed in this study, in each step, the element in feature vector x with the lowest absolute averaged weight in the corresponding element of weight vector w is removed. When the recursion terminates, the SVM model with the optimal features and the corresponding error rate are returned.

SVM Algorithm: DTI-based bmTBI Classification Algorithm

Input: Feature Matrix F, F has size |Subjects| × |Features|, Label Y

Output: Classification model M (subset of features, subset of metrics and the weight of the features and bias for the SVM classification)

Normalize F such that every column in F has zero mean and unit-variance

p = Number of the diffusion metrics

Create table T with $2^p - 1$ rows and p columns (enumerate all possible combinations of the metric set)

Fill table T with p-digit binary expression from integer 1 to $2^p - 1$

Encode each diffusion metric with an integer number ranging from 1 to p

for k = 1; k < 2^p; k++ do (test each combination of metric set)

F = {f ∈ F.column, and f.encoded_metric = 1 at row k in table T }

E_k, M_k = Routine Stepwise SVM Feature Selection (F, Y)

M = M_k if M is empty or E_k < E

E = E_k if E is empty or E_k < E

end

end

Routine Stepwise SVM Feature Selection (with a given subset of metrics)

Input: Feature Matrix F, F has size |Subjects| × |Features|, Label Y

Output: Classification model bestM, Error Er

N = the number of features (each feature is a vector with respect to each fiber track. The vector contains the values with respect to a subset of metrics)

Create vector E with size 1 × |Features|

for k = N; k > 0; k − − do

    [M(k), E(k)] = Routine SVM Cross-Validation(F,Y)

    i = argmin |M.weight| /* identify the least contributing feature */

    Delete column i from F /* remove the least contributing feature*/

end

Er = min(E)

bestM = the model in M with the minimum error

end

Routine SVM Cross-Validation

Input: Feature Matrix F, F has size |Subjects| × |Features|, Label Y

Output: Model Md, Error Er, Sensitivity Ss, Specificity Sp

Create cell M with size 1 × 10000

Create vectors E, SS, SP with size 1 × 10000

for i = 0; i < 10000; i + + do

    Randomly select 5 subjects as validating targets

    F_{train}, Y_{train} = the unchosen subjects' features, labels

    M (i) = Routine SVM (F_{train}, Y_{train})

    E(i), Ss(i), Sp(i) = Evaluate M(i) on the 5 validated subjects

    /* 5 is around 10–15% of the number of subjects in this study */

end

Md = mean(M); Er = mean(E); Ss = mean(SS); Sp = mean(SP)

end

Routine SVM

Input: Feature Matrix F, F has size |Subjects| × |Features|, Label Y

Output: Model M

Build Linear Kernel Support Vector Machine M that can optimally classify Y with L_2 regularization // Run fitcsvm on MATLAB R2018b

end

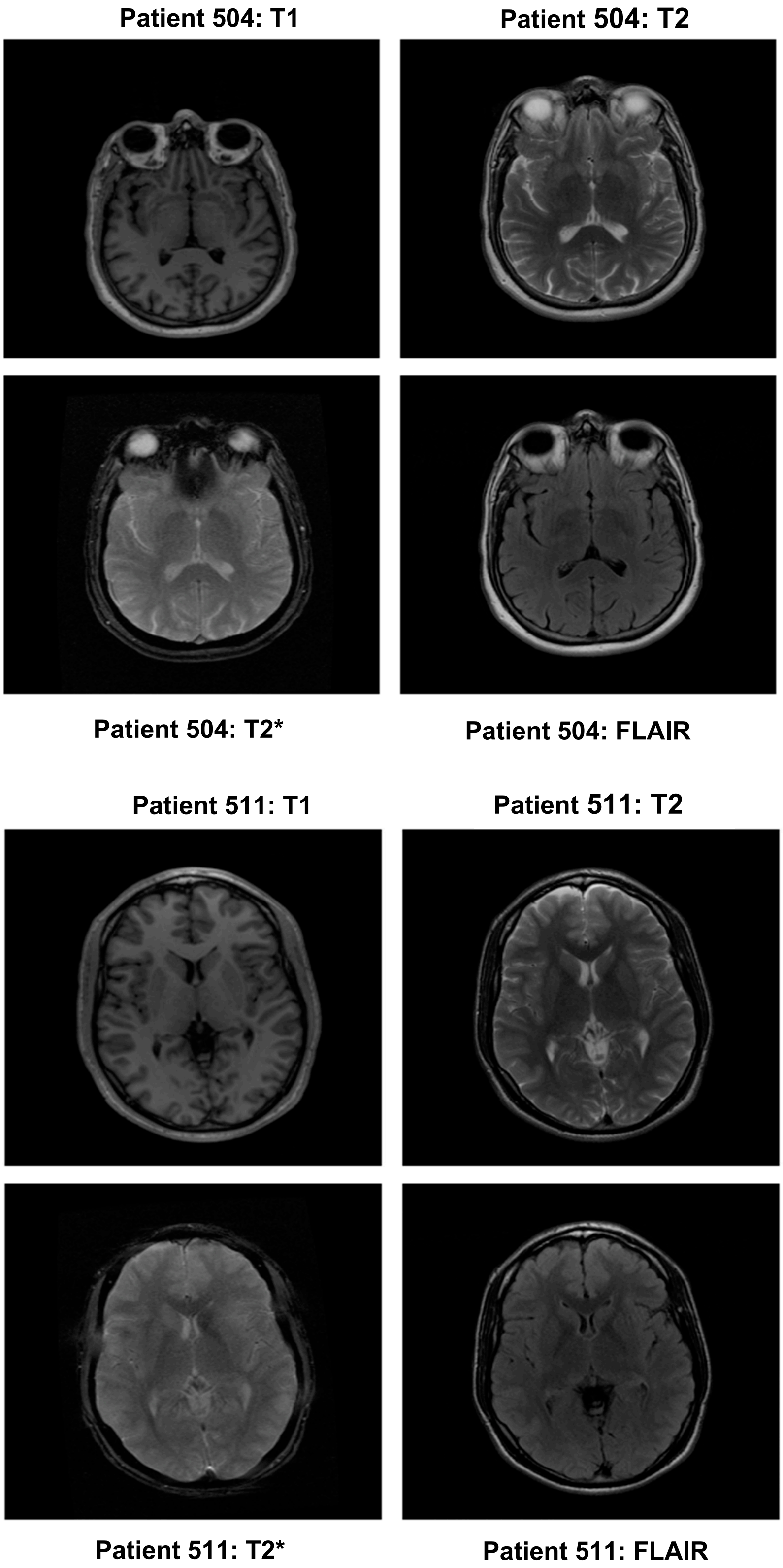

Supplementary Figure S1. Conventional anatomical MRI sequences for two patients.

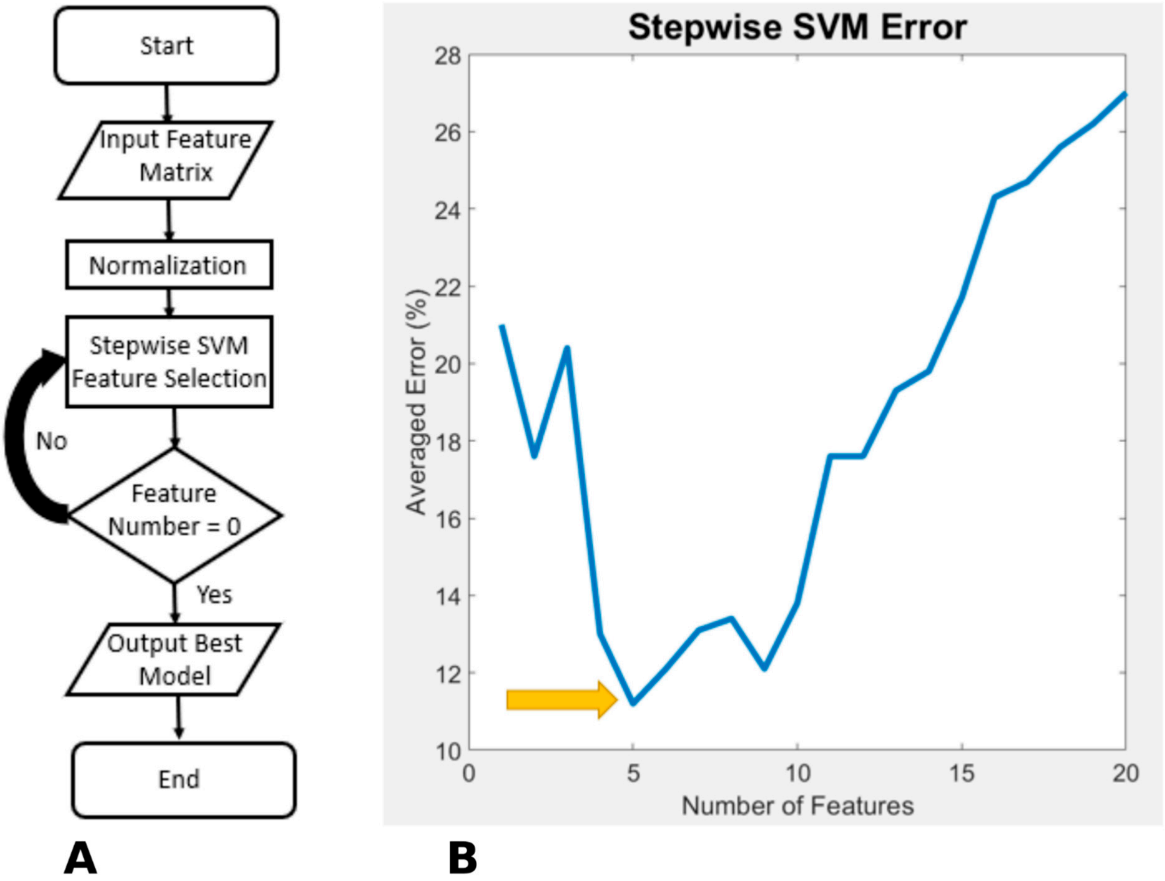

Supplementary Figure S2. Supervised vector machine (SVM) learning methodology. (**A**) The figure illustrates the procedure for the machine learning approach employed in this study. First, the FA skeleton was created with recommended TBSS procedures. Next, for each diffusion metric (FA, MD, AD, RD, and AD/RD ratio) the voxels selected were the intersection of significant clusters within the FA map by Monte Carlo group statistics and the ICBM-DTI-81 white-matter labels atlas. A feature was created for each significant cluster within each white-matter atlas location and averaged for each MRI metric. Then, all selected features were entered into our SVM algorithm. Upon completion of the algorithm, the optimal combination of the features was returned along with the accuracy, sensitivity, and the specificity of the SVM model. (**B**) The graph shows the result of recursively removing the least contributing feature in *DTI-based bmTBI Classification Algorithm.* The horizontal axis is the number of features, and the vertical axis is the averaged percentage error of the SVM model with the yellow-colored arrow indicating the best SVM model with the observed lowest error.

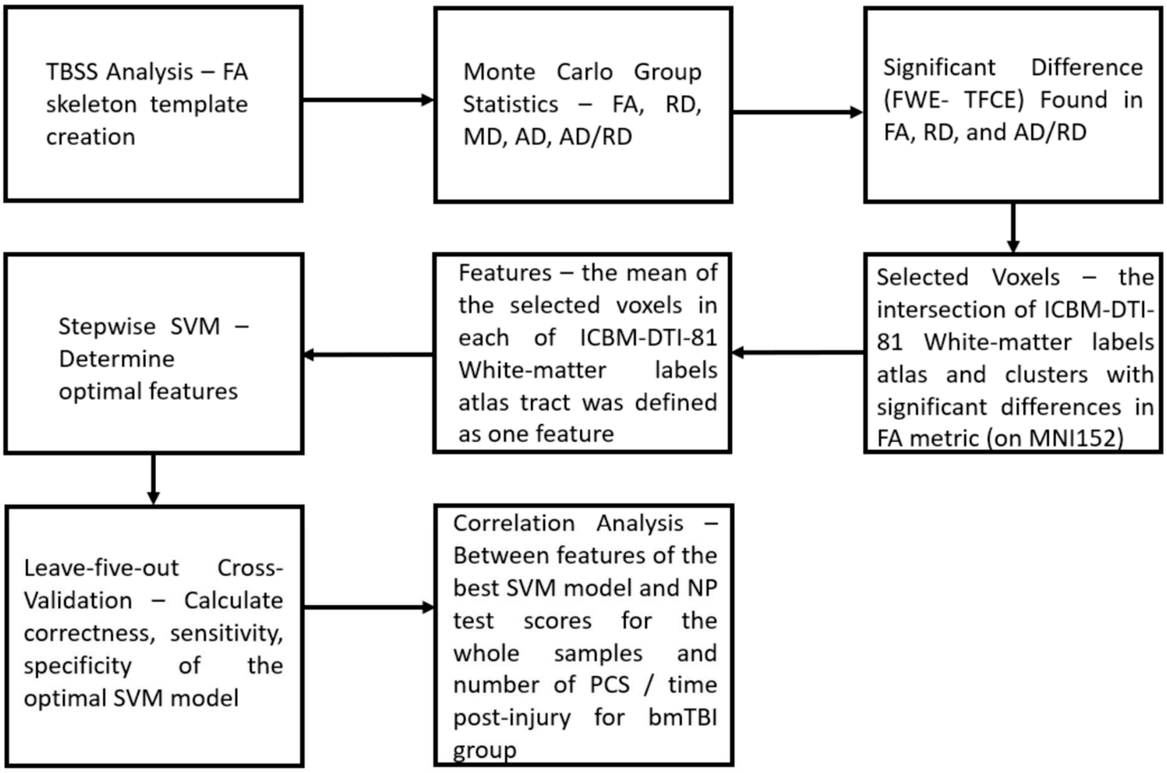

Supplementary Figure S3. Schematics of the supervised vector machine (SVM) analysis procedure in this study. The figure demonstrates the schematic of the entire SVM procedure. Standard track-based spatial statistics (TBSS) analyses were conducted on FA, RD, MD, AD, and AD/RD diffusion metrics. First, using threshold free cluster enhancement (TFCE) and Monte Carlo permutation testing, group differences in clusters of FA were identified. AD, RD, MD, and AD/RD ratio metrics were then registered to the FA skeleton on MNI152 standard space and voxel-wise statistical tests for group differences in these metrics were conducted. Next, DTI features that showed group differences are entered into our *DTI-based bmTBI Classification Algorithm,* and the best SVM model is returned. Last, correlation analysis is conducted between the features in the best SVM model and the neuropsychological (NP) scores for the combined groups and the number of PCS / time post-injury for the bmTBI group.
